# A conversation with Elizabeth Blackburn and Viviane Tabar

**DOI:** 10.1172/JCI161661

**Published:** 2022-06-01

**Authors:** Inés Fernández Maestre, Yanyang Chen, Brianna Naizir, Ushma S. Neill

**Affiliations:** Memorial Sloan Kettering Cancer Center, New York, New York, USA.

While great strides have been made with regard to gender equity in biomedical academia, there remains a stubborn imbalance in representation in senior leadership roles. The graduate students from the Gerstner Sloan Kettering Graduate School have launched a series called *The Roots of Change: Conversations about Women’s Empowerment* to grapple with the issue of representation. They invited two giants in medicine to reflect on their lives in medicine: Viviane Tabar and Elizabeth Blackburn. Dr. Tabar (Figure 1) is the chair of the Department of Neurosurgery at Memorial Sloan Kettering Cancer Center and leads a stem cell biology lab focusing on the development of human embryonic stem cell–derived dopaminergic neurons for the treatment of Parkinson’s disease, among other cell-based therapies for the repair of brain injuries. Dr. Blackburn (Figure 1) is the former president of the Salk Institute for Biomedical Studies and, before that leadership position, had a long career on the faculty at the University of California, Berkeley, and UC San Francisco. She is best known for her scientific work on telomeres; she shared the 2009 Nobel Prize in Physiology or Medicine for the discovery of telomerase. The full interview is available on the *JCI* website at www.jci.org/videos/cgms The first hour of the *Roots of Change* conversation, with feminist icon and writer Gloria Steinem, is available on the Sloan Kettering website (https://www.mskcc.org/watch-conversations-about-women-s-empowerment).

*JCI*: Both of you were born and raised outside the US. Can you start by telling us where and how you were brought up and what you were like as children? Was there anything or anyone that influenced you to become a scientist or surgeon-scientist?

Elizabeth Blackburn: I grew up in Tasmania, which is about as remote as you can be. I lived in a smallish town that was rather typically suburban. A lot of my worldview came from reading; TV wasn’t a big feature of our lives until I got a bit older. The wide world that I discovered through reading stimulated my imagination and frankly, I found it a lot more interesting than my immediate surroundings.

I grew up in a big family: my parents were both family physicians, and I was one of seven siblings. Having a lot of siblings meant I didn’t have to go out and make many friends because there were always my brothers and sisters for companionship. I could read and do things that I was interested in — like nature and animals. Having a mother who was a family physician made me think that there was no other way that a woman would have a life except with a job and a family.

I went to an all-girls school, which had certain advantages to it, even though there was a track for women who wanted to become secretaries or who the school thought should become secretaries. I remember a teacher from another school in whom I confided my interest in going into science; they said, “What’s a nice girl like you doing going into science?” I couldn’t believe it, but it had the advantage that it made me really mad, which turned into a powerful motivator. Also, I read a biography about Marie Curie when I was in middle school, written by her daughter. It captivated me because it encouraged me to think science is a wonderfully interesting thing, but also it captured a person who seemed just as passionate about being a mother. I hadn’t fathomed the tribulations that she had gone through as a woman in science. That book and her life made me think science was an interesting and worthwhile thing to be doing.

Viviane Tabar: I grew up in Beirut, Lebanon, at a time when violence and survival anxiety were really the prevalent sentiment. I was just about old enough to recognize a sense of loss when the war started. I had difficulty in visualizing the future because we were living day by day — there was a very high chance that exams the next day would be cancelled because there was a bombing. It drove me to read a lot and to develop tunnel vision. That has never left me: I’m capable of concentrating intensely. I had an intense curiosity about how living creatures were made and function. I received a gift of a microscope when I was 10 or 11 years old and looked at water — paramecium and creatures of that nature. I never really imagined that I would or could be a scientist or a physician, but my father was a great inspiration and was always extremely supportive. As the war continued, I had to leave the country if I wanted to obtain any further education or career. After my medical studies, I landed in the US, where many people opened doors for me and facilitated my career.

I was delighted to hear Elizabeth reference Marie Curie because she also was an incredible influence and inspiration to me. Not only because she earned two Nobel Prizes herself, but also because she was a single mom after Pierre Curie died. She was a nonconformer in every other way too. She had to fight for lab space; she was harassed for who she fell in love with. She was also a great humanist: she drove a primitive x-ray machine around to take radiographs of soldiers during WWI, saving many lives. She was a larger-than-life figure with formidable integrity.

*JCI*: We know that both of you have worn and still wear many hats: lab PIs, leadership roles, clinical duties in Dr. Tabar’s case, and both of you are mothers. How do you have it all? And do you think this is a question asked only of women?

Tabar: We don’t have it all. We earn it and we work for it. The juggling is an exercise in management of what is really an unstable system. It’s not easy, but if you come into this field, you should be prepared to be the CEO of your own life. You can’t be a neurosurgeon — on call for 24 hours every third day — without having a supportive partner and, if raising a child, you must be superbly organized. Also, you don’t have it all at the same time. You must recognize from the start that nothing about this is simple and arm yourself with everything that works: asking for help, being prepared, and being smart about how to manage various anxieties.

If we do not continue to maintain the push for women to be successful, we’re going to go backwards. This current great movement for hiring with an eye toward promoting gender and racial diversity among scientists and physicians is exciting: I’m seeing more people making steps and not just talking the talk. But I am concerned because we have not yet evolved our system to nurture these new hires and guarantee their success. If you were born in Africa and started your career there or in remote corners of Australia or the Middle East and you come into a system, we can’t just say, “Sink or swim! Here you are in our system, see what you can do.” We must have the courage to change things to fit different needs and backgrounds.

Blackburn: At any one moment, there’s not balance. You have to create time to intently do something that you care about. In a family of seven children with a mother who worked, it wasn’t like she had to be with us every single second telling us what to do. She and we had to organize our own time. We did just fine. For me, when our son was born, I had to stop and think what was most important and what could I cut out. The time I spend with my family is the most worthwhile time. I could miss out going to movies and plays until my son grew up — other than seeing some pretty lousy children’s movies. And eating out — I went to lunch with seminar speakers, but never dinner. You have to determine what are the things that matter in the time you’re in; there will be times when your work has higher demands and you need to sacrifice time outside of work.

Life moves on. Now that I’m Professor Emeritus, I can do things that are important to me that I didn’t have time to do before. Balance takes place over long periods of time. Every week is probably not going to be balanced.

*JCI*: In your scientific careers and leadership positions, if you experienced gender bias, how did you overcome it?

Tabar: Neurosurgery is typically a male specialty; I was never mentored by a woman, and it was incredibly difficult to get into this field. Women born and educated in the US have a challenging enough time getting into neurosurgery, but as an immigrant woman, it was a long, arduous path. The path to becoming department chair was largely an individual personal journey. There were macroaggressions that have now morphed more into microaggressions, but gratifyingly, there were no true obstacles in the way. Many of those aspects are a lot better now, albeit not completely gone. A critical element in deciding to take on a leadership position was in reaching a point where I was very excited about supporting others and building and innovating with fresh talent — being the engine in the background.

Blackburn: My path to leadership went from not knowing how to express things to feeling more comfortable saying what was on my mind and making myself heard. What I especially noticed early on was that when I said something around a table it wouldn’t be heard, but then a male colleague would say the same thing and it would resonate. Now when this happens, I say, “I’m so glad you agreed with what I just said.” Or “Thanks for supporting me in this.”

I have adopted tunnel vision, although I never thought of it quite that way, but instead, let’s say “strategic denial,” which means I gravitate toward what I do really well — science. Yes, I have been a leader, but always with the grounding in science. I’ve tried more and more to support women colleagues. Our women trainees very often undervalue their abilities compared with those of their male colleagues.

Tabar: There’s also increasingly collaborative science. We need to encourage and be mindful of helping people and celebrating their contributions.

Blackburn: Yes! Always be generous. You can be generous without diminishing yourself. Don’t denigrate the contributions of others. Set a standard; behave well to your professional colleagues. And when they’re not behaving well to you, you don’t have to be a bad example in dealing with it.

*JCI*: What led to the confidence you have now?

Blackburn: I was terribly nervous about getting tenure at University of California, Berkeley. After the procedure had gone on and I hadn’t heard anything, I figured I needed to start job hunting. I stopped into the acting chairman’s office saying I’d better start looking for new jobs, and he sort of looked at me, totally unconscious that I might have been worried about this, and said,“Oh no you don’t! It’s just sitting on some desk in the university hierarchy.” But honestly, I had no idea what he thought of me. It made me braver in my science.

The other important moment for me relates to my postdoctoral mentor. At some point, when I was in the process of applying for jobs, I realized I had no idea what he thought of me. Important for letters he’d need to write, so I asked. He said I was a first-class scientist, and I had no idea he thought that. I certainly didn’t have a lot of confidence, and that helped tremendously to hear. So it’s really important to tell your trainees that they have abilities because we don’t lift them up enough. I dread being judged, and we have this weirdly high set of standards as women. Maybe I was afraid to fail. I don’t know what it was, but I certainly didn’t have a lot of confidence.

Tabar: I see confidence as a dynamic state. I think there are milestones in your career, like becoming a professor or getting a major grant, that boost confidence. One of my pet peeves is the preponderance of lack of confidence among women scientists and trainees. If you want to be successful in science, the first step is for you to accept that it is a challenging career, and you have to be passionate. We set these impossible standards of perfection and any performance below that, unacceptable. We don’t want to ask a question unless we’re 100% sure it’s an incisive one. We don’t want to take risks, and that perpetuates a vicious cycle. You have to accept that some days you’re going to be more confident than others. I didn’t wake up feeling very confident every day when I was younger. That’s for sure.

*JCI*: What career path do you think you would have pursued if you were not a scientist or physician-scientist?

Tabar: I would have loved to be a classical pianist. I used to have this fantasy that it is the best job in the world. I might have otherwise been a writer so that I would have no bosses, no hierarchy, and full control. But those are fantasies and in truth, I’m very happy being a physician-scientist.

Blackburn: Being a pianist was exactly what I was going to say. I was a lousy player and luckily self-aware enough to realize that I wasn’t good enough; it would have been a very, very short career.

If I was shifted to a different time, perhaps I would have been a neuroscientist because I’m so fascinated by the enormous challenge the human brain presents to us.

## Figures and Tables

**Figure 1:**
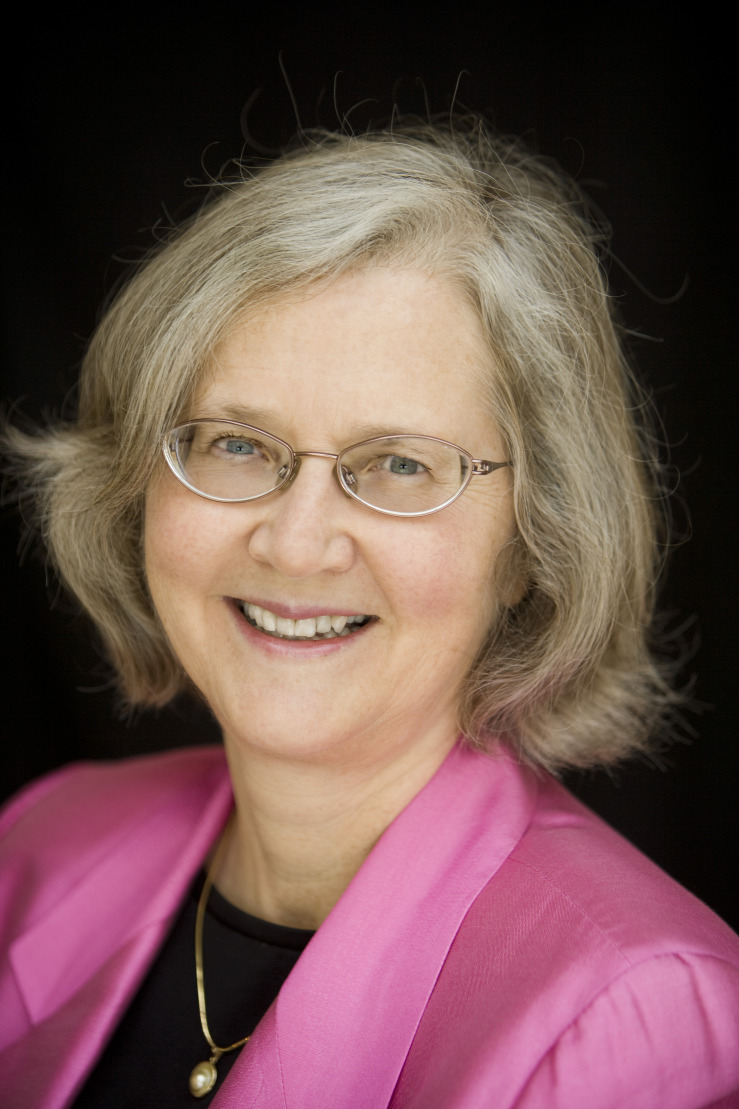
Viviane Tabar (left; image credit: Memorial Sloan Kettering) and Elizabeth Blackburn (right; image credit: Pelletier).

## Version 1 06/01/2022

Electronic publication

